# Unusual Case of Calculus in Floor of Mouth: A Case Report

**DOI:** 10.5005/jp-journals-10005-1172

**Published:** 2012-12-05

**Authors:** Rakesh N Bahadure, Nilima Thosar, Eesha S Jain

**Affiliations:** Lecturer, Department of Pedodontics and Preventive Dentistry, Sharad Pawar Dental College, Nandora, Sevagram, Wardha, Maharashtra- 442102, India, e-mail: mdsrakesh_pedo@yahoo.co.in; Professor, Department of Pedodontics and Preventive Dentistry, Sharad Pawar Dental College, Wardha, Maharashtra, India; Postgraduate Student, Department of Pedodontics and Preventive Dentistry, Chhatrapati Shahuji Maharaj Medical University, Lucknow Uttar Pradesh, India

**Keywords:** Supragingival calculus, Surgical removal, Biochemical analysis

## Abstract

Calculus consists of mineralized bacterial plaque that forms on the surfaces of natural teeth. It is supragingival or subgingival depending upon its relation with gingival margin. The two most common locations for supragingival calculus are the buccal surfaces of maxillary molars and lingual surfaces of mandibular anterior teeth. It is very important to rule out the predisposing factor for calculus formation. In the present case of an 11-year- old female child, 1.2 × 1.5 cm large indurated mass suggestive of calculus in the left side of floor of mouth was observed. After surgical removal, along with indurated mass, an embedded root fragment was seen. Biochemical analysis of the specimen detected the calcium and phosphate ions approximately equals to the level in calculus. Thus, we diagnosed it as a calculus. Oral hygiene instructions and regular follow-up was advised.

**How to cite this article:** Bahadure RN, Thosar N, Jain ES. Unusual Case of Calculus in Floor of Mouth: A Case Report. Int J Clin Pediatr Dent 2012;5(3):223-225.

## INTRODUCTION

The term ‘calculus’, which came to be used, in the 18th century, for accidental or incidental mineral buildups in human and animal bodies, like kidney stones and minerals on teeth.^1^

Saliva from the parotid gland flows over the facial surfaces of upper molars through Stenson’s duct, whereas the orifices of Wharton’s duct and Bartholin’s duct empty onto lingual surfaces of the lower incisors from the submandibular and sublingual glands,^2^ results in formation of calculus in poor hygiene cases.

Supragingival calculus consists of inorganic (70-90%)^3^ and organic components. The inorganic portion consists of 75.9% calcium phosphate, 3.1% calcium carbonate, CaCO_3_; and traces of magnesium phosphate, Mg_3_(PO_4_)_2_ and other metals.^4^ The principal inorganic components are calcium, 39%; phosphorus, 19%; carbon dioxide, 1.9%; magnesium, 0.8%; and trace amounts of sodium, zinc, strontium, bromine, copper, manganese, tungsten, gold, aluminum, silicon, iron and fluorine.^5^ At least two-thirds of the inorganic content is crystalline in structure,^6^ as hydroxyapatite, approximately 58%; magnesium whitlockite, approximately 21%; octacalcium phosphate, approximately 12%; brushite, approximately 9%. Hydroxyapatite and octacalcium phosphate are detected most frequently in 97% to 100% of all supragingival calculus constituting its bulk. Dental calculus, salivary duct calculus and calcified dental tissues are similar in inorganic composition.^2^ Present case shows the unusual location of calculus in left side of floor of mouth along with presence of embedded residual deciduous root fragment of primary mandibular first molar.

## CASE REPORT

**Fig. 1:**
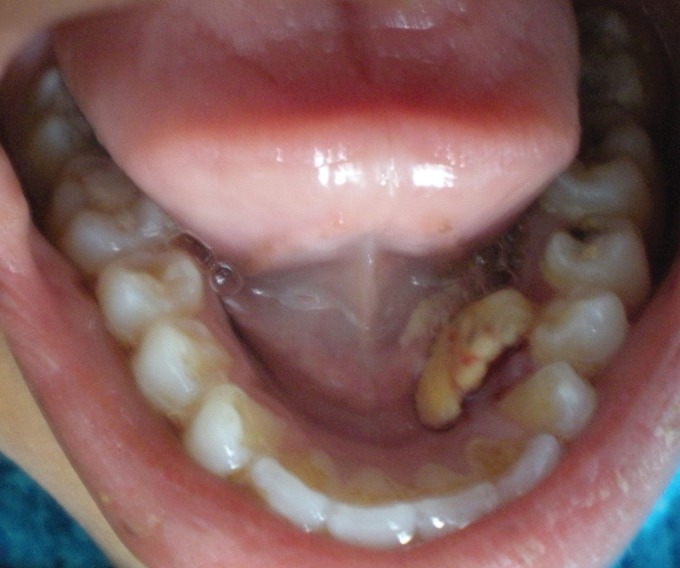
Hard indurated mass on the floor of mouth

An 11-year-old female child reported to the Department of Pedodontics and Preventive Dentistry with chief complaint of discomfort and heaviness in the lower left side of floor of mouth since 1 month. On intraoral examination, a 1.2 by 1.5 cm indurated mass in the left side of floor of mouth was seen (Fig. 1). It resembled like a calculus. It was an unusual location of calculus extending from distal surfaces of permanent left lateral incisor to the distal surface of permanent left first premolar. Superiorly it extended from gingival margin of permanent premolars and inferiorly up to floor of mouth. The mass was oval in shape. Color of hard calcified mass was yellowish. Also yellowish coating was seen posterior to the hard mass in the vestibule of tongue. There was no swelling, inflammation, infection in surrounding adjacent mucosal surfaces as well as teeth. All permanent teeth were sound without carious involvement and gingival and periodontal diseases. Patient’s oral hygiene was fair. A slight supragingival calculus was observed on the lingual surfaces of lower left premolars. Also a yellowish coating adherent to floor of mouth was seen posterior to hard calcified mass. Medical history was noncontributory.

**Fig. 2:**
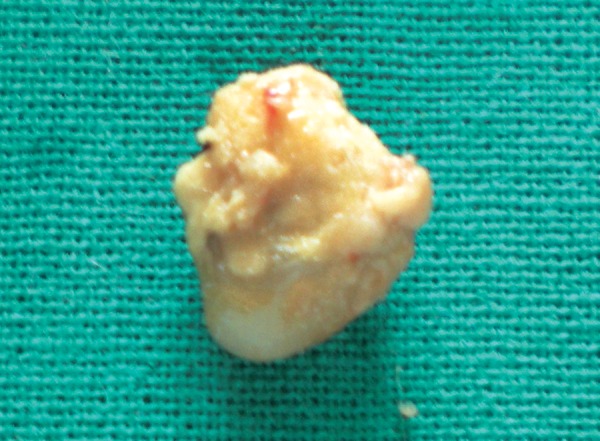
Anterosuperior view of mass

**Fig. 3:**
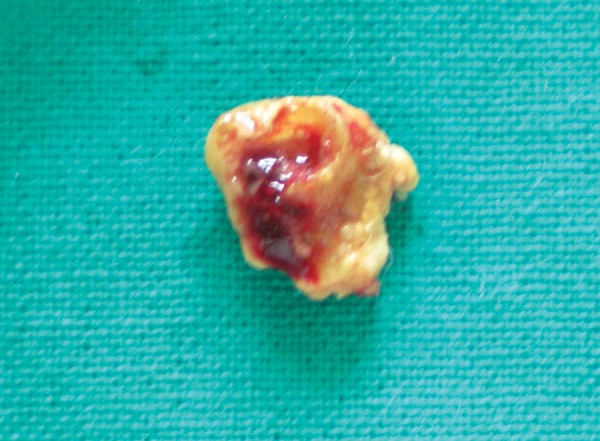
Posteroinferior view of mass

**Fig. 4:**
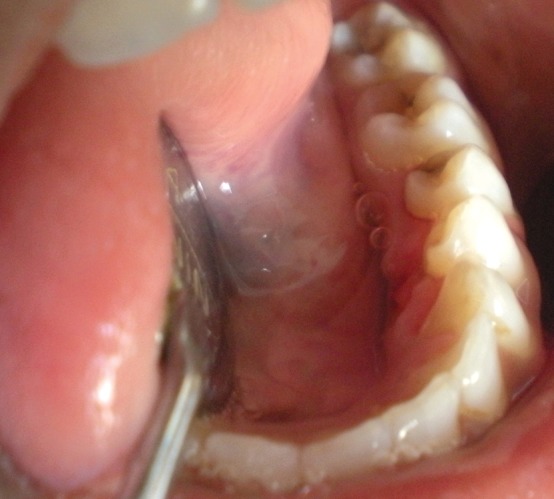
Floor of mouth after excision of mass

Patient’s blood sample was sent for routine blood investigation and to evaluate calcium and phosphate levels in serum. Under all aseptic precautions and local anesthesia, a hard calcified mass was surgically excised. Excised specimen showed presence of yellowish hard mass anterosuperiorly (Fig. 2) and embedded residual root fragment postero- inferiorly (Fig. 3). Floor of mouth appeared normal after excision of mass (Fig. 4). Specimen was sent for biochemical examination. Blood report showed serum calcium 11.02 mg% (normal range in females: 8.5-10.5 mg%), inorganic phosphate 3.72 mg% (normal range in females: 2.5 to 4.5 mg%). Biochemical report of specimen showed calcium 41.3%, inorganic phosphate 19.97% and calcium phosphate 77.02 %. Depending upon biochemical report and structure of hard calcified mass, diagnosis of calculus was considered. Patient was advised for regular recall visits and instructed for maintenance of oral hygiene.

## DISCUSSION

Calculus is a dental plaque that undergoes mineralization. The soft plaque is hardened by precipitation of mineral salts between 1st and 14th days of plaque formation. Fifty percent mineralization occurs in 2 days and 60 to 90% in 12 days. Saliva is the source of mineralization for supragingival calculus.^2^ Two principal categories to explain mineralization of plaque are:^7^

1. Mineral precipitation resulting from a local rise in degree of saturation of calcium and phosphate ions, by means of following mechanisms:
  - An increase in the pH of saliva causes precipitation of calcium phosphate salts by lowering the precipitation constant. The pH may be elevated by loss of carbon dioxide and the formation of ammonia by dental plaque bacteria or by protein degradation during stagnation.
  - Colloidal proteins in saliva bind calcium and phosphate ions and maintain a supersaturated solution with respect to calcium phosphate salts. With stagnation of saliva, colloids settle out, and the supersaturated state is no longer maintained, leading to precipitation of calcium phosphate salts.
  - Phosphatase liberated from dental plaque, desquamated epithelial cells, or bacteria precipitates calcium phosphate by hydrolyzing organic phosphates in saliva, thus increasing the concentration of free phosphate ions. Esterase is another enzyme that is present in the cocci and filamentous organisms, leukocytes, macrophages, desquamated epithelial cells of dental plaque. Esterase may initiate calcification by hydrolyzing fatty esters into free fatty acids. The fatty acids form soaps with calcium and magnesium that are later converted into the less soluble calcium phosphate salts.
2. Seeding agents induce small foci of calcification that enlarge and coalesce to form a calcified mass. Seeding agents in calculus formation are not known. It is suspected that the intercellular matrix of plaque plays an active role.^8^ The carbohydrate-protein complexes may initiate calcification by removing calcium from the saliva (chelation) and binding with it to form nuclei that induce subsequent deposition of minerals.^9^

In the present case, slight supragingival calculus was observed on the lingual surfaces of lower left premolars and yellowish coating adherent to floor of mouth was observed posterior to hard calcified mass which could be because patient could not maintain proper oral hygiene due to presence of calcified mass in that region. Rest of the teeth were clean without presence of any supra- or subgingival calculus. After excision, embedded residual root fragment was seen. So the presence of residual root fragment could be the predisposing factor for the deposition of calcified mass in lower left posterior region. The root fragment was seen in vicinity of first premolar area. So it might be the root fragment of deciduous first molar.

Since, the ductal opening of submandibular salivary gland was near to the calculus, further deposition of calcium and phosphate ions was possible in the present case. Also the submandibular salivary gland has thick, viscous salivary secretion which also can be considered as the reason for getting more and more deposition of calculus in floor of mouth.

Submandibular calculi are very rare in children and several cases have been described since in otherwise healthy children.^10^ Present case is an unusual case showing calculus in floor of mouth with embedded residual root fragment of deciduous first molar. Because of unusual location of calculus, this case can be considered as unusual case.

## CLINICAL SIGNIFICANCE

Early diagnosis of predisposing factor for initiation of calculus is very important. In the present case, presence of embedded residual root fragment of deciduous first molar was the predisposing factor for initiation of calculus formation and further mineralization of it was possible due to its location near to the ductal opening of left submandibular gland. Periodic recall visits of patient not only for supervising the maintenance of oral hygiene, but also to check for presence of any residual root pieces of deciduous molars and other predisposing factors are also important. If proper attention is not paid to such predisposing factors, it may be prone to have calcified masses in oral cavity due to mineral deposition.
